# Kisspeptin-8 Induces Anxiety-Like Behavior and Hypolocomotion by Activating the HPA Axis and Increasing GABA Release in the Nucleus Accumbens in Rats

**DOI:** 10.3390/biomedicines9020112

**Published:** 2021-01-25

**Authors:** Katalin Eszter Ibos, Éva Bodnár, Zsolt Bagosi, Zsolt Bozsó, Gábor Tóth, Gyula Szabó, Krisztina Csabafi

**Affiliations:** 1 Department of Pathophysiology, Faculty of Medicine, University of Szeged, H-6720 Szeged, Hungary; dobo.eva@med.u-szeged.hu (É.B.); bagosi.zsolt@med.u-szeged.hu (Z.B.); csabafi.krisztina@med.u-szeged.hu (K.C.); 2 Department of Medical Chemistry, Faculty of Medicine, University of Szeged, H-6720 Szeged, Hungary; bozso.zsolt@med.u-szeged.hu (Z.B.); toth.gabor@med.u-szeged.hu (G.T.); 3 Office of International Affairs, Budapest Campus, McDaniel College, H-1071 Budapest, Hungary; gyula.szabo.prf@mcdaniel.hu

**Keywords:** kisspeptin, anxiety, locomotion, Kiss1 receptor, HPA axis, HPG axis, nucleus accumbens

## Abstract

Kisspeptins (Kp) are RF-amide neuropeptide regulators of the reproductive axis that also influence anxiety, locomotion, and metabolism. We aimed to investigate the effects of intracerebroventricular Kp-8 (an N-terminally truncated octapeptide) treatment in Wistar rats. Elevated plus maze (EPM), computerized open field (OF), and marble burying (MB) tests were performed for the assessment of behavior. Serum LH and corticosterone levels were determined to assess kisspeptin1 receptor (Kiss1r) activation and hypothalamic-pituitary-adrenal axis (HPA) stimulation, respectively. GABA release from the nucleus accumbens (NAc) and dopamine release from the ventral tegmental area (VTA) and NAc were measured via ex vivo superfusion. Kp-8 decreased open arm time and entries in EPM, and also raised corticosterone concentration, pointing to an anxiogenic effect. Moreover, the decrease in arm entries in EPM, the delayed increase in immobility accompanied by reduced ambulatory activity in OF, and the reduction in interactions with marbles show that Kp-8 suppressed exploratory and spontaneous locomotion. The increase in GABA release from the NAc might be in the background of hypolocomotion by inhibiting the VTA-NAc dopaminergic circuitry. As Kp-8 raised LH concentration, it could activate Kiss1r and stimulate the reproductive axis. As Kiss1r is associated with hyperlocomotion, it is more likely that neuropeptide FF receptor activation is involved in the suppression of locomotor activity.

## 1. Introduction

The *KISS1* gene was discovered as a novel metastasis-suppressor in human melanoma cells in 1996 in Hershey, named after the famous chocolate of the city, Hershey’s Kisses [1].

*KISS1* encodes a 145-amino-acid propeptide, from which kisspeptin-54 (Kp-54) is cleaved. The proteolytical cleavage of this 54-amino-acid peptide results in shorter biologically active products, designated kisspeptin-14 (Kp-14), kisspeptin-13 (Kp-13) and kisspeptin-10 (Kp-10) [2]. Mammalian kisspeptins belong to the family of RF-amide peptides, as they carry the characteristic, conserved carboxyl-terminal Arg–Phe–NH_2_ sequence [3].

The canonical receptor of kisspeptins is a G protein-coupled receptor, Gpr54, that is fully activated by all biologically active products of the *Kiss1* gene [4]. Although Gpr54 was initially described in 1999 as an orphan receptor similar to galanin receptors [5], after being deorphanized in 2001, it was designated kisspeptin-1 receptor (Kiss1r) [6].

Upon activation of the Gα_q/11_-coupled Kiss1r, phospholipase C (PLC) is activated, leading to inositol 1,4,5-trisphosphate (IP3)-mediated intracellular Ca^2+^ mobilization. Moreover, the activation of protein kinase C (PKC) and the Gα_q_-independent recruitment of β-arrestins result in the phosphorylation of several mitogen-activated protein kinases (MAPKs), including extracellular signal-regulated kinases 1/2 (ERK1/2) and p38 [4]. MAPKs in turn regulate gene expression and induce long-term alterations in a wide range of biological processes [7], including progesterone secretion [8], trophoblast adhesion [9], and glucose-induced insulin secretion [10].

Kisspeptins also bind and activate both neuropeptide FF receptors (NPFFR1 and NPFFR2) [11]. As NPFF receptors are coupled with Gα_i/o_, their activation inhibits cAMP production. The Gβγ heterodimer released from G_i/o_ proteins was found to inhibit voltage-gated Ca^2+^ channels. Moreover, it is capable of potentiating G_q_ signaling via physical interaction with PLC [3].

Kisspeptin is expressed in several regions of the rat central nervous system, including hypothalamic nuclei [e.g., arcuate nucleus, anteroventral paraventricular nucleus (AVPV)], thalamic nuclei, the amygdala, hippocampus, lateral septum, the bed nucleus of stria terminalis, striatum, nucleus accumbens (NAc), periaqueductal grey, and locus coeruleus [12,13]. Likewise, Kiss1r has been localized in rats in the hypothalamus (e.g., paraventricular, arcuate and supraoptic nucleus), thalamus, hippocampus, amygdala, septum, striatum, raphe nuclei, and cortex [5,14].

The expression of NPFF1 receptor mRNA has been detected in the lateral septum, in thalamic and brainstem nuclei, as well as in the ventral tegmental area (VTA), NAc, the bed nucleus of the stria terminalis, the amygdala and hippocampus. NPFF2 receptor mRNA expression has been reported in thalamic nuclei, in the hypothalamus, hippocampus, VTA, the A5 noradrenergic cell group and also in the dorsal horn of the spinal cord [15,16].

Following the original discovery of its metastasis suppressor role in melanoma [1], the anti-metastatic activity of kisspeptin has been found in a variety of tumors, including bladder, ovary, colorectal, pancreas, pituitary, prostate and thyroid cancer [17].

The involvement of kisspeptin in reproduction has been a topic of extensive research since it was discovered in 2003 that kisspeptin is a potent stimulator of gonadotropin secretion [18]. The role of kisspeptin in the regulation of puberty is underlined by the finding that various loss-of-function mutations of *KISS1R* and *KISS1* are associated with isolated hypogonadotropic hypogonadism, whereas activating mutations result in central precocious puberty [19]. Hypothalamic *Kiss1* neuron populations are responsible for the regulation of the estrous cycle by mediating positive and negative feedback of gonadal steroids on gonadotropin secretion [20]. The sexually dimorphic *Kiss1* neuron population of the AVPV is responsible for the positive feedback of estrogen, thus it contributes to the surge-like secretion of GnRH. However, pulsatile GnRH secretion is regulated by the KNDy neurons (coexpressing kisspeptin, neurokinin B, and dynorphin) of the arcuate nucleus that mediate the negative feedback of estrogen [21]. Compelling evidence has suggested that KNDy neurons in the arcuate nucleus function as a major integrator of various modifiers of the reproductive axis, including metabolic signals, olfactory clues, and circadian rhythm [22,23,24,25].

Similarly to other members of the RF-amide family [26], kisspeptin has also been implicated in the regulation of nociception [27]. In a recent study, Kp-13 lowered the nociceptive threshold in mice, decreased the analgesic effect of morphine, diminished morphine tolerance and caused mechanical hypersensitivity [28].

Based on the expression of *Kiss1* and *Kiss1r* in limbic brain structures [29,30], several studies have investigated the behavioral effects of kisspeptin.

An antidepressant-like effect of kisspeptin has been reported in rats [31], and intravenous kisspeptin has also decreased negative mood in human subjects [32].

Kisspeptin neurons in the rostral periventricular area of the 3rd ventricle (RP3V) seem to regulate sexual behavior in rodents, as they are activated by male urinary odors in female mice and facilitate copulatory behavior in a NO-dependent pathway [33,34].

An interplay between kisspeptin and the hypothalamic-pituitary-adrenal (HPA) axis was suggested in 2009, when Kinsey-Jones et al. discovered that stress-induced elevation of plasma corticosterone suppresses hypothalamic kisspeptin signaling in rodents [35]. Since that time, several studies have been conducted with controversial results.

In paraventricular nucleus-derived cell lines, Kp-10 increased the gene expression of arginine vasopressin (AVP) and oxytocin, while suppressing the expression of corticotropin releasing hormone (CRH). However, it failed to influence the activity of the HPA axis in vivo, as intraperitoneally (ip.) administered Kp-10 had no effect on plasma corticosterone and adrenocorticotropic hormone (ACTH) levels in rats [36]. Likewise, kisspeptin administration had no effect on anxiety in human subjects [32].

In 2013, our group reported an anxiogenic effect of intracerebroventricularly (icv.) administered Kp-13 in rats. Kp-13 not only induced a significant increase in plasma corticosterone level, but also decreased the number of entries into the open arms and the time spent in them in the elevated plus maze test. Moreover, it has stimulated spontaneous locomotion and it also had a hyperthermic effect lasting for several hours after treatment [37].

An anxiogenic property of kisspeptin signaling has also been proposed by the experiments of Delmas et al., in which *Kiss1r* KO mice have spent more time in the open arms in the elevated plus maze test, indicating a suppression of anxiety. The most pronounced anxiolytic effect was observed when kisspeptin signaling in GnRH neurons was selectively rescued in *Kiss1r* KO animals, suggesting a modulatory role of gonadal steroids. Interestingly, no significant effect of *Kiss1r* KO was detected on the behavioral parameters of the open field test [38].

In zebrafish, however, the central administration of kisspeptin has been associated with an anxiolytic tendency in the novel-tank diving test and a significantly reduced fear response to alarm substance [39].

In a recent study, a Cre-dependent, stimulatory DREADD (Designer Receptors Exclusively Activated by Designer Drugs) viral construct has been targeted to the *Kiss1* neurons of the posterodorsal medial amygdala (MePD) in mice. Upon selective activation of MePD *Kiss1* neurons by clozapine-N-oxide, a significant increase in open arm exploration has been observed in the elevated plus maze, suggesting an anxiolytic role of this neuron population [40].

There are several possible explanations for the ambiguous results reported in the literature. On one hand, the route of administration could be a determining factor. Peripheral administration of Kp has failed to influence the activity of the HPA axis in rats (0.13 µg/µL Kp-54 ip.) [36] and the activity of the limbic system in human subjects (1 nmol/kg/h Kp-54 iv. over 75 min) [32]. In contrast, central Kp-13 (1 or 2 µg icv.) had a pronounced anxiogenic effect in rats [37]. It is likely that the doses applied by Rao et al. [36] and Comninos et al. [32] were too low to exert an anxiogenic effect. In their investigation into the effect of peripheral or central Kp administration on the reproductive axis in rats, Thomson et al. have found that 1 nmol of icv. Kp-10 was sufficient to significantly raise plasma luteinizing hormone (LH) concentration, but a 100-fold dose was required for the same effect in case of ip. treatment [41]. Likewise, the selective activation of MePD *Kiss1* neurons [40] points to the function of a distinct neuron population, whereas central kisspeptin treatment [37] reflects a general central effect by activating neurons bearing Kiss1r throughout the brain.

On the other hand, the differences could also be attributed to the variety of species involved in these experiments. The kisspeptin system of zebrafish is strikingly different from the mammalian one, both in terms of anatomy and function [39,42], thus the results of studies on zebrafish should be interpreted with caution.

Some studies have also reported that kisspeptin might play a role in the regulation of locomotor activity. Icv. Kp-13 has induced an increase in not only spontaneous, but also in exploratory locomotion in male Sprague-Dawley rats [37]. In line with these results, Tolson et al. have found that *Kiss1r* KO female mice exhibit decreased locomotor activity and energy expenditure, leading to obesity [43].

It has been discovered that kisspeptin attenuates morphine effect [28], and is expressed in the NAc [44], pointing to its possible involvement in the regulation of mesocorticolimbic dopaminergic activity. Interestingly, the centers of reward and addiction have also been implicated in the regulation of locomotion. First, quinpirole (a D2 receptor agonist) injected into the NAc has suppressed exploratory locomotion in rats [45], whereas bicuculline (a GABA_A_ receptor antagonist) administration into the nucleus induced hyperactivity with prolonged exploratory behavior in rats [46]. Second, the selective activation of dopaminergic neurons in the VTA by DREADD has induced a pronounced and sustained hyperactivity in rats, which effect could be reproduced by activating selectively activating the dopaminergic pathway between the VTA and NAc [47]. Thus, it is possible that kisspeptin might influence locomotion by modulating the activity of the VTA or NAc.

Nowadays kisspeptin analogs and antagonists are attracting considerable attention due to their potential therapeutic use in various gynecological conditions, including infertility, polycystic ovary syndrome and precocious puberty [48]. The shortest natural bioactive form of kisspeptin is the 10 amino acid long Kp-10, which has higher affinity to Kiss1r than Kp-54 [49]. According to molecular docking studies, ASN4, SER5, GLY7, ARG9 and PHE10 of Kp-10 are involved in the formation of hydrogen bonds between the peptide and Kiss1r [50]. Consequently, shorter kisspeptin fragments containing these amino acids might be able to bind and possibly activate the receptor.

The aim of the current study was to investigate whether the 8 amino acid long fragment of kisspeptin is capable of influencing the behavior of rats similarly to kisspeptin. Following icv. treatment with Kp-8, elevated plus maze (EPM), computerized open field (OF), and marble burying (MB) tests were performed. Serum corticosterone and luteinizing hormone concentrations were measured to assess the activation of the HPA axis and Kiss1 receptors, respectively. Moreover, dopamine release from the VTA and NAc and GABA release from the NAc were measured using ex vivo superfusion to further characterize the mechanism of action.

## 2. Materials and Methods

### 2.1. Animals and Housing Conditions

Adult male Wistar rats (Domaszék, Csongrád, Hungary) weighing 150–250 g were used for the experiments at the age of 6–8 weeks. The animals were housed under controlled conditions at constant room temperature, with a 12–12-h light dark cycle (lights on from 6:00 a.m.). The rats were allowed free access to commercial food and tap water.

The animals were kept and handled during the experiments in accordance with the instructions of the University of Szeged Ethical Committee for the Protection of Animals in Research, which approved these experiments. Permission for the experiments (number: X./1207/2018, date: 6 July 2018.) has been granted by the Government Office of Csongrád County Directorate of Food Chain Safety and Animal Health. Each animal was used for only one experimental procedure.

### 2.2. Intracerebroventricular Cannulation

A stainless steel Luer cannula (10 mm long) was implanted in the right lateral cerebral ventricle for icv. administration. The cannula was inserted under sodium pentobarbital (Euthasol, Phylaxia-Sanofi, 35 mg/kg, ip.) anaesthesia, according to the following stereotaxic coordinates: 0.2 mm posterior and 1.7 mm lateral to the bregma, and 3.7 mm deep from the dural surface [37]. Subsequently, it was secured to the skull with dental cement and acrylate. The experiments started after a recovery period of 1 week. All experiments were carried out between 8:00 a.m. and 10:00 a.m.

### 2.3. Peptide Synthesis

Kisspeptin-8 (WNSFGLRF-NH2) was synthesized on a Rink Amide MBHA resin (Bachem, Bubendorf, Switzerland, subst.: 0.52 mmol/g) using *N*^α^-9-Fluorenylmethoxycarbonyl (Fmoc) protected amino acids (IRIS Biotech GmbH, Marktredwitz, Germany) by manual solid phase peptide synthesis by the Department of Medical Chemistry (University of Szeged). The resin was swollen in dichloromethane (DCM). The Fmoc group was removed by treating the peptide-resin with 20% piperidine/*N*,*N*-dimethylformamide (DMF) solution twice (5 + 15 min). Solvents were purchased from VWR (Radnor, PA, USA).

The amino acids were activated with *N*,*N*′-dicyclohexylcarbodiimide and 1-hydroxybenzotriazole in 50% DCM/DMF. The peptide-resin was incubated with this mixture for 3 h. The resin was washed with DMF (3×) and DCM (3×) after the deprotection and coupling steps.

The assembled peptides were cleaved from the resin by treating it with the following cleavage cocktail for 3 h: 90% trifluoroacetic acid (TFA) (Pierce, Rockford, IL, USA), 5% water, 2% dithiotreitol, 2% triisopropylsilane.

**Figure A1:**
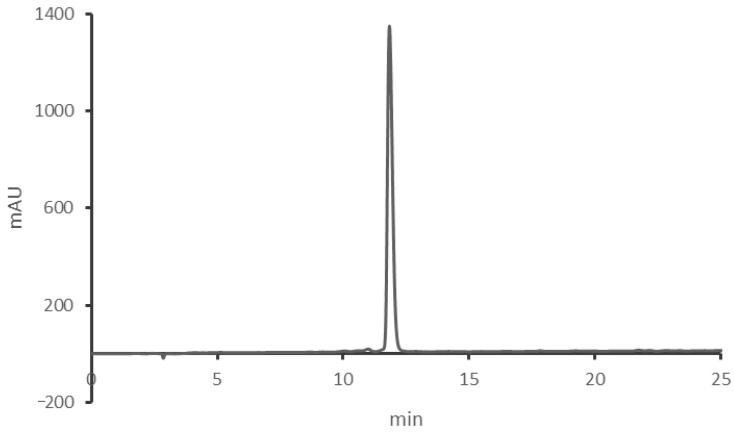
HPLC trace of Kisspeptin-8. Column: Phenomenex Luna C18, 5 μ, 100 Å, 4.6 mm × 250 mm, flow rate: 1 mL/min, wavelength: 220 nm, A eluent: 0.1% TFA in water, B eluent: 0.1% TFA/80% ACN/water, gradient: 30–55% eluent B in eluent A over 25 min.

**Figure A2:**
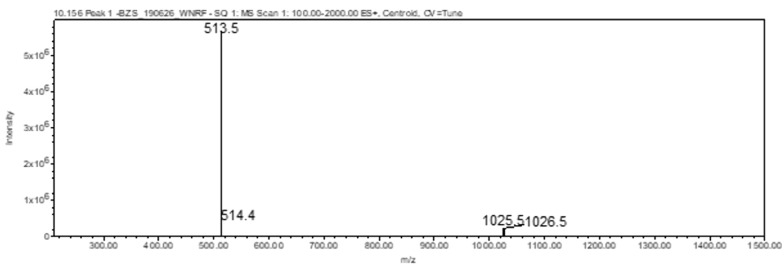
The ESI-MS trace of Kp-8 peptide.

The peptides were precipitated with diethyl ether, dissolved in a mixture of acetonitrile (ACN) and water and lyophilized. The crude peptides were analyzed by HPLC (Hewlett-Packard Agilent 1100 system, column: Luna, c18 (2), 250 × 4.6 mm, 5 µm, 100 Å, Phenomenex, Aschaffenburg, Germany) and ESI-MS. The peptides were purified on a preparative HPLC column (Phenomenex Luna, c18 (2), 250 × 21.2 mm, 10 µm, 100 Å) using a Shimadzu 20-LC system. The fractions were analyzed on the above mentioned analytical HPLC system and measured by electrospray ionization mass spectrometry (ESI-MS) (see Appendix A Figure A1 and Figure A2 for the results). The pure fractions were pooled and freeze-dried.

### 2.4. Treatment

The rats were treated icv. in a volume of 2 µL over 30 s using a Hamilton microsyringe (Merck KGaA, Darmstadt, Germany). The doses applied were 0.1 or 1 µg of Kp-8 dissolved in 0.9% saline. Control animals were injected with 2 µL of 0.9% saline alone. The animals were treated 30 min prior to the behavioral tests. Collection of trunk blood for LH ELISA, corticosterone ELISA and serum corticosterone measurement were carried out 15 min and 30 min after icv. treatment, respectively.

### 2.5. Behavioral Tests

#### 2.5.1. Elevated Plus Maze Test

The EPM apparatus is a plus-shaped platform 50 cm above the ground. The maze consists of four arms (50 cm × 10 cm each): two opposing open arms and two closed arms enclosed by a 10 cm high wall. The test is based on two conflicting motivations of rodents: to avoid open, brightly lit spaces and to explore novel environment. The avoidance of open arms reflects anxiety-like behavior [51]. 30 min after icv. treatment the rats were placed in the maze facing one of the open arms, then their behavior was recorded by a camera suspended above the maze for 5 min. The time spent in each arm, as well as the number of entries per arm were registered by an observer blind to the experimental groups. The percentage of entries into the open arms and the percentage of time spent in the open arms were also calculated. The experiments were conducted between 8 a.m. and 10 a.m. and the apparatus was cleaned with 96% ethyl-alcohol after each session.

#### 2.5.2. Computerized Open Field Test

The novelty-induced locomotor activity of rats was assessed using the Conducta 1.0 System (Experimetria Ltd., Budapest, Hungary). The system consists of black plastic OF arenas (inside dimensions: 48 × 48 cm, height: 40 cm) with 5 horizontal rows of infrared diodes on the walls to register both horizontal and vertical locomotion. The center of each box is illuminated by a LED lightbulb (230 lumen) from above the box. The central zone of the arena is defined as a 24 × 24 cm area in the center of the box. 30 min after icv. treatment the rats were placed in the center of the box and their behavior was recorded by the Conducta computer program for 60 min. Six behavioral parameters were measured during the experiment: total time and total distance of ambulation, immobility time, number of rearings (vertical locomotion), time spent in the central zone (central area of 24 × 24 cm), and distance travelled in the central zone. The OF experiments were conducted between 8 a.m. and 10 a.m. and the apparatus was cleaned with 96% ethyl-alcohol after each session.

#### 2.5.3. Marble Burying Test

MB is a regularly used paradigm for the assessment of anxiety-like and compulsive-like behavior [52]. Our protocol was based on the method described by Schneider and Popik [53]. The animals were removed from their plexiglass home cages (420 × 275 × 180 mm) and temporarily moved into another cage before the experiment. Meanwhile the home cage was prepared for the experiment by increasing the depth of bedding material to 5 cm. Following icv. treatment one animal was placed back into the home cage for 30 min in order to acclimatize to the thick bedding. Then 9 glass marbles of 2.5 cm diameter were arranged in 3 rows along the shorter wall of the cage. The experiment was conducted for 10 min and recorded by a video camera above the cage. After the session, the animal was removed from the cage and the number of buried marbles (>50% marble covered by bedding material) was counted. The marbles were cleaned with 96% ethyl-alcohol after each session. After the experiment, the video recording was evaluated. The count and duration of two types of goal-oriented interactions with marbles (burying of marbles and moving marbles without burying them) were assessed.

### 2.6. Serum Corticosterone, Luteinizing Hormone and Total Protein Concentration Measurement

For the measurement of serum corticosterone and protein concentration, the animals were decapitated 30 min after icv. treatment. For the assessment of serum LH, decapitation was performed 15 min after icv. treatment. Trunk blood was collected into test tubes and left at room temperature for 30 min to clot, then it was centrifuged for 10 min at 3500 rpm. The samples were stored at −80 °C until the assays were performed. Serum corticosterone concentration was measured using a competitive corticosterone ELISA kit (Cayman Chemical, Ann Arbor, MI, USA), according to the manufacturer’s instruction. Serum LH concentration was determined using a sandwich LH ELISA kit (Wuhan Xinquidi Biological Technology Co., Wuhan, China), according to the manufacturer’s instructions. The Pierce Coomassie (Bradford) Protein Assay Kit (Thermo Fisher Scientific, Waltham, MA, USA) was used, according to the manufacturer’s instructions for the measurement of total serum protein concentration. The absorbance was measured at 595 nm with a NanoDrop One^C^ microvolume spectrophotometer (Thermo Fisher Scientific, Waltham, MA, USA).

### 2.7. Ex Vivo Superfusion

Before the ex vivo superfusion, the animals did not undergo icv. cannulation. The rats were rapidly decapitated, and their brains were removed from the skull. Dissection was performed with the help of a brain matrix, a tissue puncher and razor blades, on a filter paper moistened with phosphate-buffered saline, on top of a Petri dish filled with ice. The NAc was removed from both sides, following the method of isolation described by Heffner [54]. The VTA was isolated as described by Salvatore et al. [55]. The tissue was cut to 300 µm slices and incubated for 30 min in 5 mL of Krebs solution (Reanal, Hungary) bubbled with carbogen gas (5% CO_2_ and 95% O_2_). Then 5 μL of [3H]GABA (PerkinElmer Inc., Waltham, MA, USA) was added to the NAc and 5 μL of [3H]Dopamine (PerkinElmer Inc., Waltham, MA, USA) was added to the VTA or the NAc. Afterwards the slices were transferred evenly into the four cylindrical chambers of the superfusion system (Experimetria Ltd., Budapest, Hungary), and superfusion with carbogen-bubbled Krebs solution was started at body temperature (37 °C). A constant flow rate of 227, 7 μL/min was maintained with a peristaltic pump (Minipuls 2, Gilson, Middleton, WI, USA). After 30 min of superfusion, the collection of superfusates into Eppendorf tubes was started with a multichannel fraction collector (FC 203B, Gilson, Middleton, WI, USA). Fractions were collected every two minutes for 32 min. At 6 min, 1 µg of Kp-8 dissolved in 1 mL of Krebs solution was added directly into the chambers. From the 12th minute of fraction collection, electrical stimulation of square-wave impulses was delivered for two minutes (ST-02 electrical stimulator, Experimetria Ltd., Budapest, Hungary). Then, the tissue from each chamber was transferred into a beaker containing 600 μL of Krebs solution for ultrasonic homogenization (Branson Sonifier 250, Emerson Electric Co., St. Louis, MO, USA).

Afterwards 3 mL of Ultima Gold scintillation cocktail (Perkin-Elmer Inc., Waltham, MA, USA) was pipetted into 4 rows of 17 scintillation vials. Subsequently, 200 μL of the 16 fractions collected and of the suspension of the tissue from the corresponding chamber were added into each row of vials. The samples were homogenized mechanically for 30 min.

The radioactivity of samples was detected with a liquid scintillation spectrometer (Tri-carb 2100 TR, Hewlett-Packard Inc., Palo Alto, CA, USA). Fractional dopamine or GABA release (FR) was calculated from the counts per minute (CPM), according to the equation below, in which *i* stands for the number of fraction and *n* = 16. *CPM_17_* refers to the CPM of the homogenized tissue sample corresponding to the fraction:

$$FR_{i}=100\cdot \frac{CPM_{i}}{4\cdot CPM_{17}+\sum_{i+1}^{n}CPM_{i}}$$

### 2.8. Statistical Analysis

Data are presented as mean + SEM. Statistical analysis and graph editing were performed using the GraphPad Prism 8 software. One-way ANOVA with Holm-Sidak’s post-hoc test was applied for the analysis of EPM results. One-way ANOVA with Dunnett’s post-hoc test was used for the analysis of cumulative OF results, as well as for the evaluation of serum corticosterone, LH and total protein measurements. Two-way RM ANOVA with Holm–Sidak’s post-hoc test was performed for the evaluation of 5-min intervals in the OF test as well as for the interpretation of dopamine and GABA release from the NAc. Mixed-effects analysis with Holm–Sidak’s multiple comparison test was performed for the evaluation of fractional dopamine release from the VTA. Kruskal–Wallis test with Dunn’s post-hoc test was performed for the analysis of MB results. Curve fitting for ELISA tests was performed according to the manufacturers’ instructions.

## 3. Results

### 3.1. Behavioral Tests

#### 3.1.1. Elevated Plus Maze

**Figure 1:**
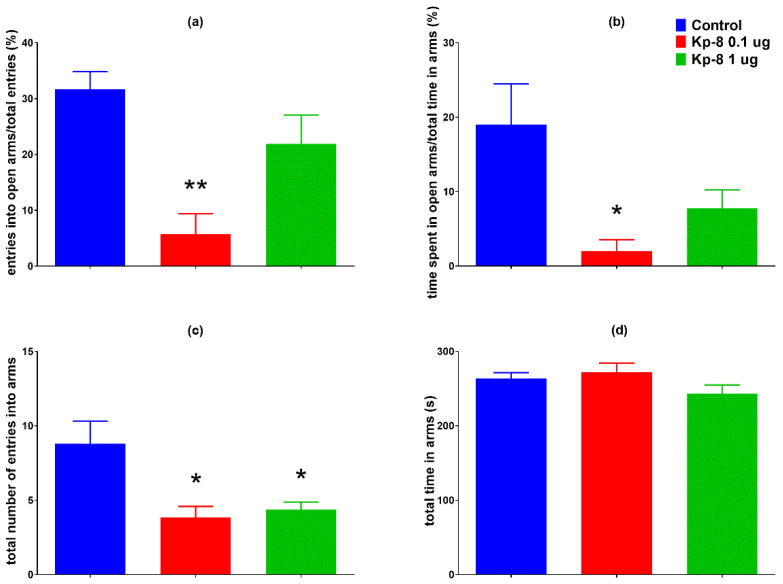
Elevated plus maze results: (**a**) percentage of entries into open arms, (**b**) percentage of time spent in the open arms, (**c**) total number of entries into arms, (**d**) total time spent in the arms of the maze, *n* = 7–9, * *p* < 0.05 vs. control, ** *p* < 0.01 vs. control.

The 0.1 µg dose of Kp-8 significantly reduced the percentage of entries into the open arms of the plus maze (Figure 1a, F (2, 20) = 9.196, *p* = 0.0007), as well as the percentage of time spent in the open arms of the maze (Figure 1b, F (2, 20) = 4.431, *p* = 0.0202). A decrease in the total number of entries into the arms was induced by both 0.1 µg and 1 µg of Kp-8 (Figure 1c, F (2, 20) = 5.927, *p* = 0.0153). There was no significant difference among the groups in the total time spent in the arms (Figure 1d, F (2, 20) = 1.932, *p* = 0.1710).

#### 3.1.2. Computerized Open Field Test

**Figure A3:**
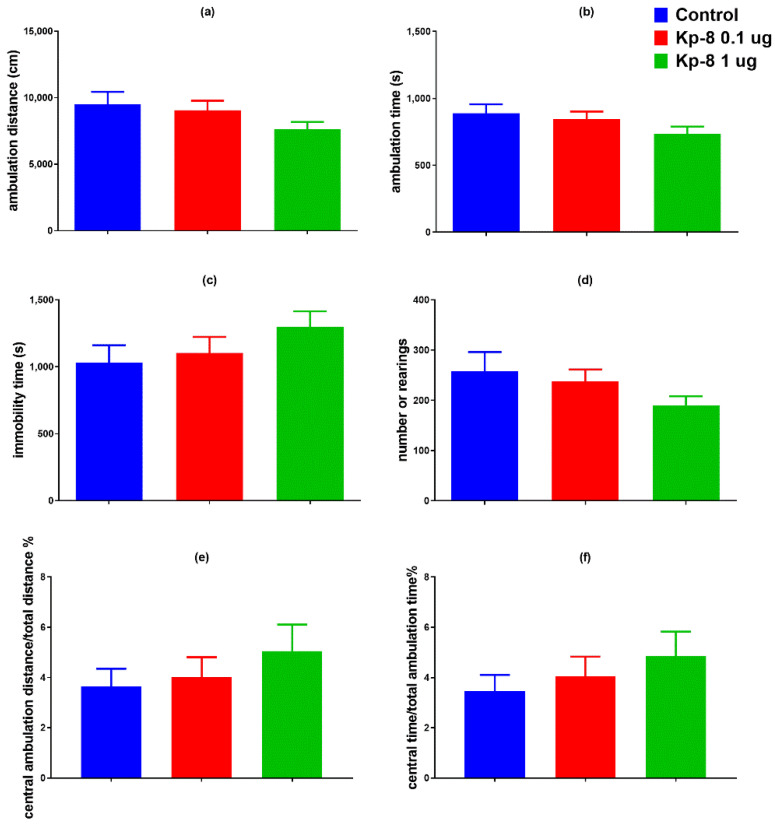
Cumulative data of 60-min open field test: (**a**) total distance of ambulation, (**b**) total time of ambulation, (**c**) total time spent immobile, (**d**) number of rearings, (**e**) percentage of distance travelled in the central zone, (**f**) percentage of time spent in the central zone, *n* = 12–13.

The cumulative results obtained after 60 min of data collection did not show any significant change in behavior (see Appendix B Figure A3).

**Figure 2:**
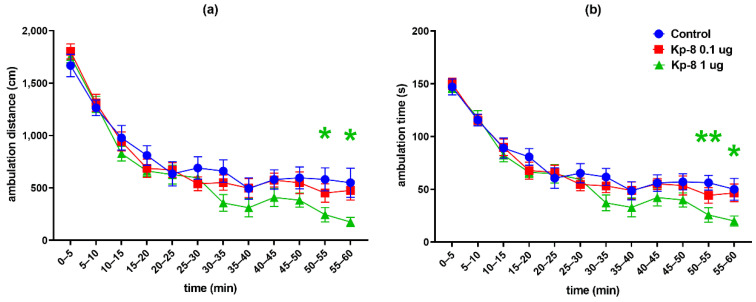
Open field test results in 5-min intervals: (**a**) total distance travelled in the arena, (**b**) total ambulation time. The color of * refers to the treatment group which significantly differs from the control group. *n* = 12–13, * *p* < 0.05 vs. control, ** *p* < 0.01 vs. control.

However, significant differences were found following the analysis of each 5-min interval. As seen in Figure 2a, the two-factor RM-ANOVA on the distance travelled in the arena revealed a significant main effect for the time factor (F (5.389, 183.2) = 113.8, *p* < 0.0001). Following a peak in the first five minutes the ambulation distance was steeply decreasing until a lower level of basal locomotor activity was reached around 30 min. The distance travelled at 50–55 and 55–60 min was lower in the 1 µg Kp-8 group than in the control group (*p* = 0.0334 and *p* = 0.0410, respectively).

Regarding total ambulation time, there was a significant main effect for the time factor (F (6.138, 208.7) = 98.03, *p* < 0.0001) with a similar pattern of steep then mild decrease (Figure 2b). The 1 µg Kp-8 group spent less time with ambulation than the control group at 50–55 min (*p* = 0.0090) and 55–60 min (*p* = 0.0326), as well.

**Figure 3:**
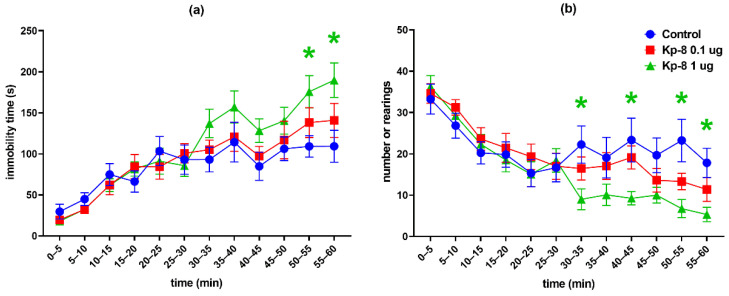
Open field test results in 5-min intervals: (**a**) total time spent immobile, (**b**) total number of rearings. The color of * refers to the treatment group which significantly differs from the control group. *n* = 12–13, * *p* < 0.05 vs. control.

The two-way ANOVA on immobility yielded a significant main effect for the time factor (F (5.396, 183.5) = 34.51, *p* < 0.0001) and interaction (F (22, 374) = 2.249, *p* = 0.0012). The time spent immobile was increasing during the experiment, showing a tendency reciprocal to that of ambulation time and distance (Figure 3a). Compared to control, the 1 µg dose of Kp-8 significantly increased immobility at 50–55 and 55–60 min (*p* = 0.0202 and *p* = 0.0186, respectively).

Considering the number of rearing sessions, a significant main effect for the time factor was detected (F (6.756, 229.7) = 7.52, *p* < 0.0001), along with a statistically significant interaction between time and treatment (F (22, 374) = 3.095, *p* < 0.0001). As seen in Figure 3b, a pronounced difference started to appear among treatment groups from 30 min. There was a significant decrease in the number of rearings in the 1 µg Kp-8 group at 30–35 min (*p* = 0.0369), 40–45 min (*p* = 0.0445), 50–55 min (*p* = 0.0182) and 55–60 min (*p* = 0.0108).

**Figure 4:**
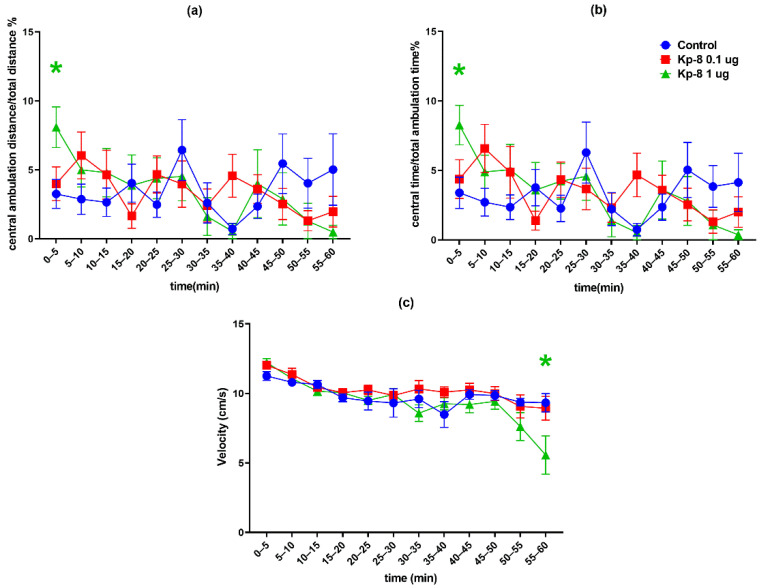
Open field test results in 5-min intervals: (**a**) percentage of distance travelled in the central zone of the arena, (**b**) percentage of time spent in the central zone of the arena, (**c**) average velocity of ambulation. The color of * refers to the treatment group which significantly differs from the control group. *n* = 12–13, * *p* < 0.05 vs. control.

Having calculated the average velocity for each timeframe, a significant main effect for the time factor (F (4.044, 129.4) = 12.17, *p* < 0.0001) and interaction (F (22, 352) = 1.940, *p* = 0.0073) could be seen, as shown in Figure 4c. There was no significant difference between treatment groups until 55 min, when the speed of the 1 µg Kp-8 group dropped (*p* = 0.0479).

Figure 4a shows the percentage of central ambulation distance, calculated by dividing the distance travelled in the central zone of the arena by the total ambulation distance, multiplied by 100. Time factor (F (6.920, 235.3) = 2.207, *p* = 0.0351) and interaction between time and treatment (F (22, 374) = 1.767, *p* = 0.0185) both significantly accounted for the variation, but there was no difference among the groups, except in the first 5 min, when the central ambulation distance of the 1 µg Kp-8 group was higher than that of control (*p* = 0.0429).

The percentage of central ambulation time was calculated by multiplying the ratio of central time and total ambulation time by 100, as shown in Figure 4b. There was a significant main effect for the time factor (F (6.981, 237.3) = 2931, *p* = 0.0059), as well as for the interaction (F (22, 374) = 1.945, *p* = 0.0070). In the first 5 min, the central ambulation time of the 1 µg Kp-8 group significantly exceeded the central time of the control group (*p* = 0.0409), otherwise there was no difference among the groups.

#### 3.1.3. Marble Burying Test

**Figure 5:**
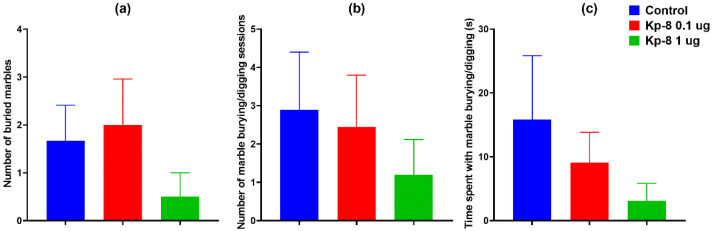
Results of marble burying test: (**a**) number of buried marbles (at least 50% covered with bedding material), (**b**) number of marble burying sessions, (**c**) duration of marble burying activity, *n* = 9–10.

There was no significant difference in the number of buried marbles among the groups (Figure 5a). Two types of goal-oriented interactions with the marbles were distinguished: marble burying and marble moving.

Marble burying is an interaction involving digging around the marbles, resulting in marbles covered with bedding material. As seen in Figure 5b,c, neither the number of marble burying sessions, nor the duration of marble burying activity changed significantly with treatment, although a tendency of reduced burying activity was observable.

**Figure 6:**
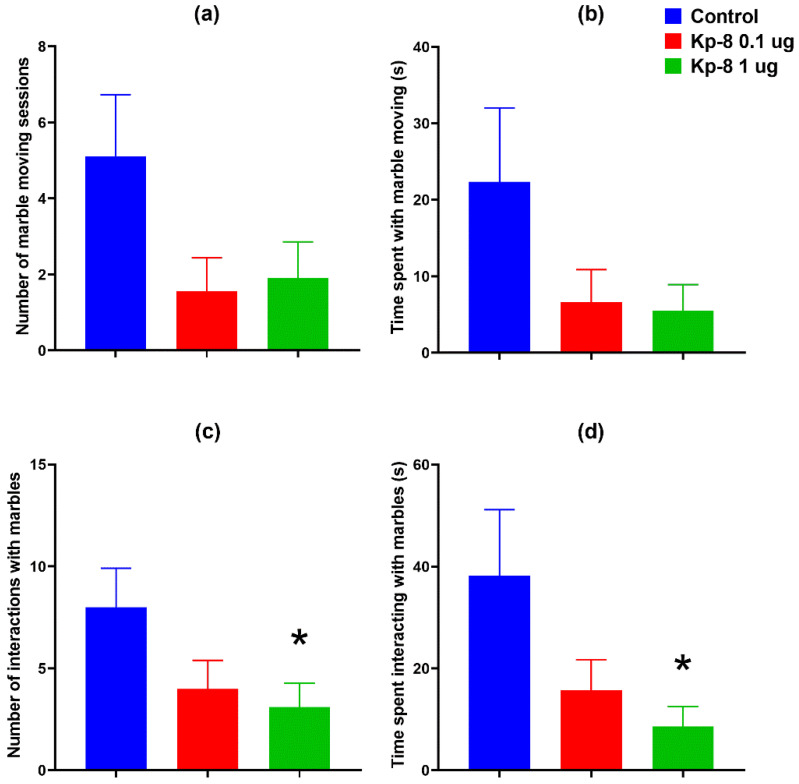
Results of marble burying test: (**a**) number of marble moving sessions, (**b**) duration of marble moving activity, (**c**) total number of interactions with marbles, (**d**) total duration of interactions with marbles, * *p* < 0.05 vs. control. *n* = 9–10.

Marble moving is an interaction that involves rolling, moving the marbles with the forelegs, without successfully covering it with bedding material. Similarly to marble burying, there was no significant difference in the number and duration of marble moving among the groups (Figure 6a,b), although a tendency of suppressed marble moving could be seen in the groups treated with Kp-8.

However, taken the two types of interactions together, the 1 µg Kp-8 group interacted with the marbles fewer times than the control group (Figure 6c, *p* = 0.0499) and they also spent less time with goal-oriented interactions with the marbles (Figure 6d, *p* = 0.0274).

### 3.2. Serum Corticosterone, LH and Total Protein

**Figure 7:**
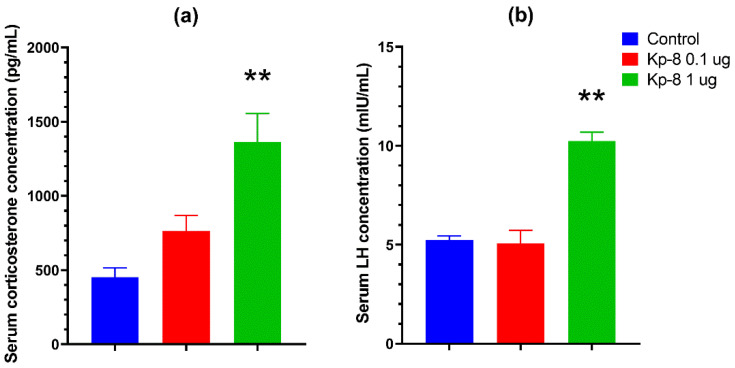
ELISA results: (**a**) serum corticosterone concentration (pg/mL), *n* = 4–5, ** *p* < 0.01 vs. control, (**b**) serum LH concentration (mIU/mL), *n* = 4–9, ** *p* < 0.01 vs. control.

The results of serum corticosterone and LH measurement can be seen in Figure 7. One-way ANOVA showed a significant effect of Kp-8 treatment both on corticosterone (F (2, 10) = 12.02, *p* = 0.0022) and LH concentration (F (2, 15) = 41.31, *p* < 0.0001). A robust increase in serum corticosterone concentration was detected 30 min after icv. treatment with 1 µg of Kp-8 (*p* = 0.001 vs. control). The 0.1 µg dose had a tendency to elevate corticosterone concentration, but the change was not significant (*p* = 0.306 vs. control). The 1 µg dose of Kp-8 also raised serum LH concentration 15 min after icv. treatment (*p* = 0.0001 vs. control), but the 0.1 µg dose had no effect on LH (*p* = 0.961 vs. control). There was no difference in serum protein concentration among the groups (F (2, 17) = 2.365, *p* = 0.124, *n* = 5–8): The mean serum protein concentrations with SD were 45.74 ± 4.898, 40.44 ± 3.115 and 45.13 ± 7.045 g/L in the control, 0.1 µg and 1 µg groups, respectively.

### 3.3. Ex Vivo Superfusion

**Figure 8:**
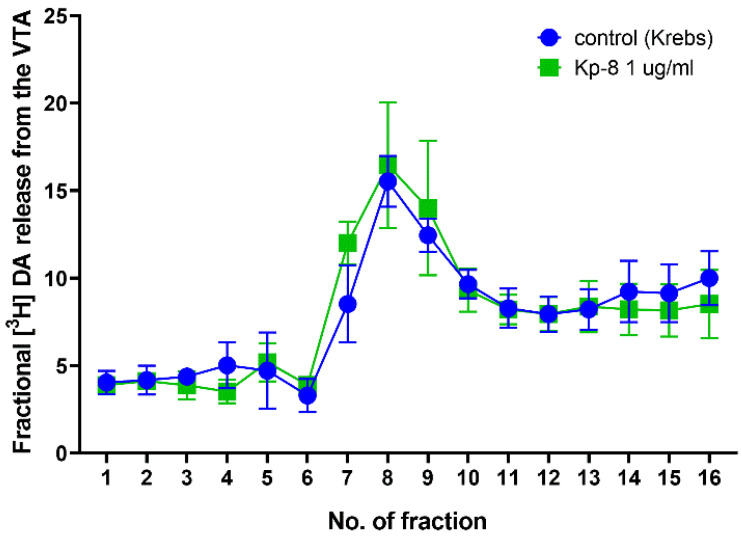
Fractional dopamine release from the ventral tegmental area. *n* = 4–5.

Figure 8 shows fractional dopamine release from the VTA. A p value was not calculated for the time factor (F (15.00, 104.0) = 16.41). There was no significant main effect neither for the treatment factor (F (1, 7) = 0.0008258, *p* = 0.9779), nor for the interaction between treatment and time (F (15, 104) = 0.5151, *p* = 0.9273). There was no significant difference between the groups at any other time point.

**Figure 9:**
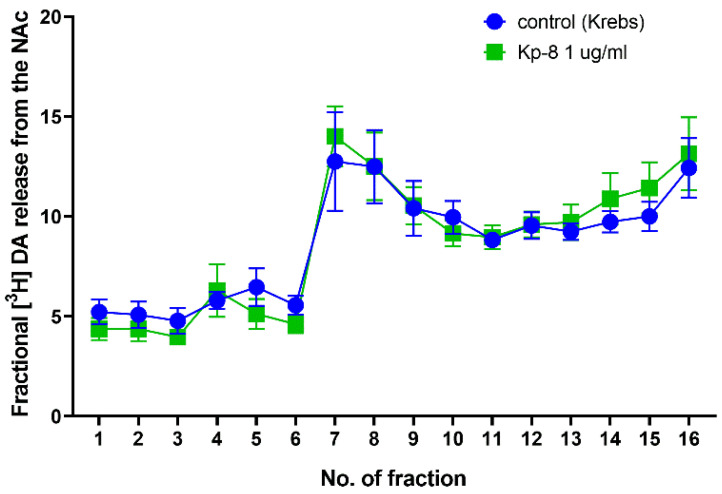
Fractional dopamine release from the nucleus accumbens. *n* = 7–8.

Likewise, Kp-8 did not influence fractional dopamine release from the NAc (Figure 9). However, there was a significant main effect for the time factor (F (3.134, 40.75) = 22.48, *p* < 0.0001). No significant main effect was found for the treatment factor (F (1, 13) = 0.0007717, *p* = 0.9783), and for the interaction between treatment and time (F (15, 195) = 0.4387, *p* = 0.9658). No significant difference could be detected at any specific time point between the groups.

**Figure 10:**
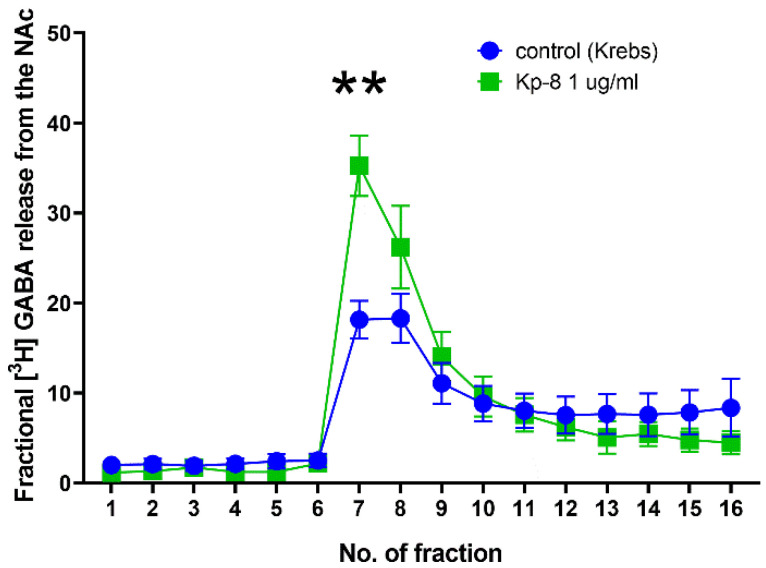
Fractional GABA release from the nucleus accumbens. ** *p* < 0.01 vs. control, *n* = 5.

As shown in Figure 10, Kp-8 increased fractional GABA release from the NAc. There was a significant main effect for the time (F (2.227, 17.82) = 60.49, *p* < 0.0001) and interaction (F (15, 120) = 7.395, *p* < 0.0001) factors. In the seventh fraction, following electrical stimulation, fractional GABA release was significantly higher from the Kp-8 treated brain slices than from the control tissue (*p* = 0.0039).

## 4. Discussion

Short kisspeptin analogs are promising candidates in the treatment of infertility and other gynecological conditions [48]. Kisspeptins exert their effect on the reproductive axis via Kiss1r [4], but they also bind to and activate NPFF1 and NPFF2 receptors with lower affinity [11]. In our study, we investigated the behavioral and biological effects of icv. Kp-8 in male rats via performing a battery of behavioral tests (EPM, OF, MB), determining serum corticosterone and LH levels, as well as measuring dopamine release from the VTA and NAc, and GABA release from the NAc.

The 0.1 µg dose of Kp-8 (but not the 1 µg dose) decreased the percentage of open arm entries and open arm time in the EPM, which is characteristic of anxiety-like behavior [51]. It is in accordance with our previous experiments in which a preference for closed arms has been observed following icv. treatment with Kp-13 [37]. Still, it must be noted that only a higher dose of Kp-13 has exerted an anxiogenic action, whereas in the case of Kp-8 an approximately 10-times lower dose was effective. The dose–response curve of Kp-8 shows a bell-shape (or inverted U-shape), that has been reported in several studies involving neuropeptides [56,57,58]. This phenomenon, when a lower dose is stimulatory, whereas a higher dose is inhibitory or ineffective, is called hormesis [59]. A review by Calabrese has reported a wide range of explanations for hormetic responses, including receptorial and intracellular mechanisms. For example, the same substance might have a stimulatory effect in a low dose, but an inhibitory effect in a high dose either via the same receptor (often mediated by a so-called ‘molecular switch’), or via different receptors to which it has higher and lower affinity, respectively [60]. Based on a review on RF-amides and their receptors, kisspeptins in general can bind to their cognant receptor, Kiss1r, and to NPFF receptors with different affinity [3], the latter of which depends on the length of the peptide: the full length Kp (in rats Kp-52) has a lower affinity to NPFF receptors, whereas the shorter endogenous derivatives’ binding affinity to NPFF receptors is higher. Furthermore, Rouméas et al. have performed systemic N-terminus deletions and benzoylations of Kp-10, which has revealed a progressive loss of affinity of the shorter fragments to Kiss1r and a conserved high affinity to NPFF receptors. In contrast, these shorter benzoylated fragments could still act as full agonists on Kiss1r, whereas on NPFF receptors a partial agonistic action has been observed [61]. How the benzoylation affects the affinity profile of these Kp-10 fragments is not known, yet it is possible that the unmodified Kp-8 also has an altered binding profile. In point of fact, agonists of NPFF1 and NPFF2 receptors have been implicated in anxiety [62,63]. Indeed, both Kiss1r and NPFF receptors could mediate the anxiety-like action of Kp-8, and we cannot rule out the possible activation of other receptors, as well.

Kp-8 also induced an elevation in serum corticosterone concentration: the 0.1 µg dose showed a tendency to increase it, whereas the 1 µg dose was significant. Corticosterone elevation is indicative of the activation of the HPA axis, the parvocellular neurons in the hypothalamic paraventricular nucleus (PVN) might have released CRH and AVP, followed by the secretion of ACTH from the pituitary, which consequently triggered the secretion of glucocorticoids from the adrenal cortex. In a study by Rao et al., Kp induced an increase in AVP mRNA expression in PVN-derived cell lines [36], and thus it is possible that Kp-8 activated the axis by increasing AVP release. Moreover, the activity of the HPA axis is modulated by limbic brain regions, including the amygdala [64]—an expression site of both Kp and Kiss1r [65]—which stimulates the HPA axis and regulates the behavioral response to stress [64,66]. The increase in glucocorticoid signaling in itself is also associated with anxiety-like behavior [67]. This result ties well with our previous study in which icv. Kp-13 has also caused an elevation in corticosterone concentration in a higher dose [37].

However, in the first 5 min of computerized open field test, the animals treated with 1 µg of Kp-8 spent more time in the central zone of the arena, which is considered a sign of anxiolysis [68]. As there was no difference in central locomotor activity at any other time point, this result should be interpreted with caution. At the beginning of the OF, the animals are placed in the center of the arena, so it is possible that the increase in central time reflects an initial latency in approaching the periphery rather than a real anxiolytic effect.

In addition, it is not uncommon to have discrepancies between the EPM and OF results. For example, chlordiazepoxide has reduced anxiety-like behavior in the EPM, but has had no significant effect in the OF in Lewis rats [69]. Although the principles of OF and EPM are similar, the two tests seem to load on different factors of anxiety [70]. Moreover, the approach of open arms in the EPM and central locomotion in the OF seem to be independently inherited in rats [71].

Altogether Kp-8 seemed to increase anxiety-like behavior and activate the HPA axis. These results are in accordance with the anxiogenic effect of icv. Kp-13 in rats [37] and the anxiolysis observed in *Kiss1r* KO mice [38]. However, Kp has not influenced anxiety in rats in the study by Rao et al. [36], which might be attributed to the peripheral route of administration and the relatively low dose of Kp used in the experiment. Likewise, intravenous Kp has had no effect on anxiety in human subjects [32]. Apart from the route of administration, another important factor to consider is the species: the Kp system of zebrafish greatly differs from that of mammals, which might explain the anxiolytic property of kisspeptin observed in the study of Ogawa et al. [39]. Moreover, when compared to a systemic treatment, the regional modulation of neuronal activity can have strikingly different consequences. For example, the selective activation of kisspeptin neurons in the medial posterodorsal amygdala has decreased anxiety [40].

The number of total arm entries in the EPM reflects the general locomotor activity of the animals [72]. Both the 0.1 and 1 µg doses of Kp-8 reduced the number of arm entries, suggesting that Kp-8 might cause hypolocomotion.

The 60-min OF also yielded some remarkable results. When placed in a novel environment, all groups exhibited a pronounced exploratory activity with intense ambulation and a high number of rearings. Following a gradual decline until approximately 30 min, activity returned to a basal level. From that point, differences have started to appear among the groups, as there was a decrease in ambulation and rearing activity, as well as an increase in immobility in the group treated with 1 µg of Kp-8. These results point to a decrease in spontaneous locomotion.

Kp-8 has also significantly reduced the number and time of goal-oriented interaction with marbles in the MB. Although MB has long been considered a test for anxiety-like behavior, now several authors have expressed doubts about it [73]. According to Thomas, the number of buried marbles does not correlate with other anxiety-like traits, namely central time in the open field test and light-dark transitions in the light-dark box test [74]. The utility of the test as a screening tool for anxiety has also been questioned based on the findings that most anxiolytics and antidepressant drugs reduce marble burying behavior secondary to drug-induced hypolocomotion [73]. It has been suggested that digging and burying are species-specific, innate behavioral patterns that are likely triggered by an exploratory drive [75] or by the bedding itself [76]. Nowadays marble burying is regarded as a sign of repetitive, compulsive-like behavior, which is highly dependent on general locomotor activity [52]. Consequently, the reduction in goal-oriented interactions with the marbles is most likely a sign of suppressed locomotion in our study.

These findings contrast with the results reported by our group on the effects of icv. Kp-13, as it has increased exploratory and spontaneous locomotion in rats [37]. In a study by Tolson et al., *Kiss1r* KO female mice have exhibited a decrease in spontaneous locomotion and energy expenditure, but the mutation has had no such effect in male animals [43], pointing to a possibly gender-dependent effect.

Icv. Kp-8 has stimulated LH release in our study, which is a sign of reproductive axis activation, secondary to Kiss1R binding and activation in the hypothalamus. Kiss1R is expressed on hypothalamic GnRH neurons [4], and upon its activation repetitive LH pulses are generated [77]. In our study, the 1 µg dose of Kp-8 caused a significant increase in LH concentration. This is in accordance with literature data as icv. injection of a similar dose of Kp-10 has exerted an LH surge [41]. It must be noted though, that 0.1 µg of Kp-8 did not affect LH release. This is not surprising since Thomson et al. have obtained a similar result when Kp-10 was administered icv in a similarly low dose [41]. Furthermore, in a study by Pheng et el., icv. administered Kp-10 at a similarly low dose was unable to stimulate LH release in male rats, only the full length Kp-52 did. The authors have postulated that slower degradation of Kp-52 might explain their results, but it is also possible that the different binding profiles of Kp-52 and Kp-10 are in the background [78]. This also might explain our result, since Kp-8 similarly to Kp-10 might bind with higher affinity to the NPFF receptors, more specifically to NPFF1 receptor. One of the ligands of NPFF1 receptor is the RF-amide-related peptide 3 (RFRP-3), which has an inhibitory effect on the reproductive axis in adult male rats [79,80]. Thus, it is possible that at a lower dose, the two opposing actions of Kp-8 result in no change in LH concentration. Nevertheless, further studies are required to determine the affinity of Kp-8 to its receptors and the degree of calcium mobilization upon receptor activation. As the hypolocomotor effect of Kp-8 seems to be in contrast with previous studies on kisspeptin and locomotion [37,43], it is likely that this effect is mediated by other mechanisms.

One possible explanation is the activation of NPFF receptors. Kp-8 has activated human NPFF2 receptors in *Xenopus* oocytes [81], and its N-terminally benzoylated form has shown to fully preserve the affinity of Kp-10 to NPFF1 and NPFF2 receptors [61], which are universally activated by all members of the RF-amide family [3].

It is noteworthy that several members of the RF-amide family have been reported to modulate locomotor activity, pointing to the possible role of NPFF receptors in the regulation of locomotion. Similarly to Kp-8, icv. treatment with RF-amide related peptide 1 (RFRP-1) has reduced total locomotor activity and has also induced anxiety-like behavior and HPA axis activation [63]. Likewise, intra-VTA injection of NPFF has reduced spontaneous locomotion in rats [82]. Interestingly, icv. NPFF has inhibited morphine-induced hyperlocomotion, but has failed to affect the locomotor activity of naïve rats [83]. Although icv. neuropeptide AF (NPAF) has also had an anxiogenic effect, contrary to RFRP-1 and NPFF, it has stimulated spontaneous and exploratory locomotion [56].

Another possible reason for the development of hypolocomotion is the modulation of the mesocorticolimbic dopaminergic system. Based on the expression of kisspeptin in the NAc, as well as the expression of NPFF1 and NPFF2 receptors in the NAc and VTA [16,44], it was reasonable to investigate whether Kp-8 has a direct effect on the VTA-NAc circuitry. The dopaminergic pathway connecting the VTA and NAc has long been implicated in the regulation of locomotion. As a matter of fact, VTA dopaminergic neurons are responsible for the locomotor-enhancing effect of cocaine [84]. Our hypothesis was that Kp-8 might suppress locomotion by directly modulating the activity of VTA dopaminergic neurons. However, in our ex vivo superfusion study, Kp-8 has not affected dopamine release from slices obtained from the VTA and NAc.

As the interaction between Kp and GABA is known from the literature [85,86], it also seemed possible that Kp-8 might directly affect GABA release in NAc. GABAergic neurons in the NAc have been shown to inhibit dopaminergic projections from the VTA [87]. In fact, GABAergic activity can also be connected with the suppression of locomotion, as locomotor activity has increased when GABA_A_ receptor antagonists were injected into the NAc core [46]. In our study, Kp-8 significantly increased GABA release from NAc slices. This result suggests that Kp-8 might directly modulate the activity of GABAergic neurons in NAc, which could contribute to the suppression of locomotor activity. It must be mentioned, however, that ex vivo superfusion measures only the direct effect of Kp-8 on live tissue slices obtained from the NAc, but the complex assessment of the whole VTA-NAc circuitry is beyond the scope of this method. Consequently, further studies (e.g., in vivo microdialysis) are required to confirm these findings on the circuit level.

When considering the receptors involved, it is possible that Kp-8 alters GABA release via NPFF1 or NPFF2 receptors, which are abundantly expressed in the VTA and the NAc, and likely involved in the modulation of both dopaminergic and GABAergic neuronal activity [16]. The role of NPFF receptors is further supported by the results of Cador et al., who have reported a decrease in novelty-induced locomotion upon intra-VTA NPFF treatment. Kiss1r expression, however, has only been detected in the NAc of humans, but not in rodents [88], so it is unlikely that Kp-8 could modulate NAc activity via Kiss1r.

Furthermore, the contribution of altered metabolism and thermoregulation should not be ruled out in the background of altered locomotor activity. Kisspeptin’s stimulatory effect on locomotion seemed to be coupled with metabolic effects in the literature. Icv. Kp-13 has induced hyperthermia [37], and *Kiss1r* KO has resulted in obesity, increased adiposity and impaired glucose tolerance in female mice [43]. Kp could also be involved in hypothalamic appetite regulation by exciting proopiomelanocortin (POMC) neurons and inhibiting neuropeptide Y/Agouti-related peptide (NPY/AgRP) neurons, resulting in an anorexigenic effect [89]. Although only a few studies have addressed the metabolic effects of other RF-amides, they have usually revealed significant results. Icv. NPFF has reduced food intake in food-deprived rats [90] and also had a hypothermic effect in mice [91]. Moreover, the stimulation of central NPFF1 and NPFF2 receptors have evoked hypothermia and hyperthermia, respectively [92].

Alternatively, Kp-8 might modulate the activity of other, locomotion-related systems differently than the naturally occurring kisspeptins, resulting in an opposing effect of locomotion. For example, central Kp-10 treatment has stimulated vasopressin release in rats [93], and vasopressin has induced hyperlocomotion by acting on V1a receptors on hypothalamic orexin/hypocretin neurons in mice [94]. Furthermore, kisspeptin has been shown to induce BDNF expression in the hippocampus [95] and the lack of active BDNF in tissue plasminogen activator deficient mice has been associated with a decrease in nocturnal wheel running activity [96]. It is a question of future research to investigate whether Kp-8 could modulate vasopressin release and BDNF secretion in a similar or different fashion as other kisspeptins.

## Data Availability

Data is contained within the article.

## Appendix A

Following solid-phase peptide synthesis, Kp-8 was analyzed using HPLC and ESI-MS, the results of which are shown in Figure A1 and Figure A2, respectively.

## Appendix B

During the computerized open field test, data were collected for 60 min, in 5-min timeframes. The cumulative results obtained after 60 min of data collection can be seen in Figure A3. There was no significant difference in any of the parameters measured: total distance of ambulation (Figure A3a, F (2, 34) = 1.691, *p* = 0.1994), total time of ambulation (Figure A3b, F (2, 34) = 1.728, *p* = 0.1928), time spent immobile (Figure A3c, F (2, 34) = 1.274, *p* = 0.2927), number of rearings (Figure A3d, F (2, 34) = 1.522, *p* = 0.2328), percentage of central ambulation distance (Figure A3e, F (2, 34) = 0.6885, *p* = 0.5092), and percentage of central ambulation time (Figure A3f, F (2, 34) = 0.7265, *p* = 0.4910).
